# Development of a New Threshold of Toxicological Concern Database of Non-cancer Toxicity Endpoints for Industrial Chemicals

**DOI:** 10.3389/ftox.2021.626543

**Published:** 2021-03-23

**Authors:** Takashi Yamada, Masayuki Kurimoto, Akihiko Hirose, Chihae Yang, James F. Rathman

**Affiliations:** ^1^Division of Risk Assessment, Center for Biological Safety Research, National Institute of Health Sciences, Kawasaki, Japan; ^2^Molecular Networks GmbH, Nürnberg, Germany; ^3^Department of Chemical and Biomolecular Engineering, College of Engineering, The Ohio State University, Columbus, OH, United States

**Keywords:** TTC database, industrial chemicals, non-cancer endpoint, provisional TDI, HESS, chemical space

## Abstract

In cases where chemical-specific toxicity data are absent or limited, the threshold of toxicological concern (TTC) offers an alternative to assess human exposure below which “there would be no appreciable risk to human health.” The application of TTC to non-cancer systemic endpoints has been pursued for decades using a chemical classification and Point of Departure (POD). This study presents a new POD dataset of oral subacute/subchronic toxicity studies in rats for 656 industrial chemicals retrieved from the Hazard Evaluation Support System (HESS) Integrated Platform, which contains hundreds of reliable repeated-dose toxicity test data of industrial chemicals under the Chemical Substances of Control Law in Japan. The HESS TTC dataset was found to have less duplication with substances in other reported TTC datasets. Each chemical was classified into a Cramer Class, with 68, 3, and 29% of these 656 chemicals distributed in Classes III, II, and I, respectively. For each Cramer Class, a provisional Tolerable Daily Intake (TDI) was derived from the 5th percentile of the lognormal distribution of PODs. The TDIs were 1.9 and 30 μg/kg bw/day for Classes III and I, respectively. The TDI for Cramer Class II could not be determined due to insufficient sample size. This work complements previous studies of the TTC approach and increases the confidence of the thresholds for non-cancer endpoints by including unique chemical structures. This new TTC dataset is publicly available and can be merged with existing databases to improve the TTC approach.

## Introduction

The Threshold of Toxicological Concern (TTC) is a pragmatic approach for risk assessment and management of chemicals of known structure with low human exposure and little or no compound-specific toxicological data. TTC uses exposure thresholds for humans derived from oral toxicity test results for cancer and non-cancer endpoints. If the exposure is lower than the TTC value, it suggests a very low potential for safety concerns, and if it exceeds the TTC value, additional data is needed to assess the risk of concern.

For non-cancer endpoints, Munro et al. (1996) proposed a comprehensive human exposure threshold based on the relationship between chemical structure and toxicity. Using a test dataset of substances including industrial chemicals, pharmaceuticals, food additives, and pesticides, as well as decision tree created by Cramer et al. (1978), 613 substances were classified into structural classes I, II, and III. The 5th percentile value was calculated from the distribution of Point of Departure (POD) values for each class, and then the human exposure value for each class was derived.

Although the Munro dataset was intended to cover a wide range of chemical space, questions have been raised whether it adequately represents chemicals and structures used in contexts other than the original application to food-related substances (Dewhurst and Renwick, 2013). Since then, the application of the non-cancer TTC approach has been extended to include a variety of chemicals in large-scale toxicity databases. For industrial chemicals, Cramer classification and TTC values were re-evaluated using the RepDose database developed by the Fraunhofer Institute of Toxicology & Experimental Medicine (Tluczkiewicz et al., 2011) and the German regulatory database of new chemicals, ELINCS (Kalkhof et al., 2012). For cosmetics-related chemicals, a new large dataset termed the COSMOS TTC dataset was constructed under the European COSMOS project of Safety Evaluation Ultimately Replacing Animal Testing (SEURAT-1). The applicability of the TTC approach and the expansion of included chemical space have been verified in detail (Yang et al., 2017). Recently, Reilly et al. (2019) tested the TTC values for food-related substances in the OpenFoodTox database constructed by The European Food Safety Authority (EFSA, 2020). The TTC approach was also proposed for antimicrobials using data based on global antimicrobial inventories (Yang et al., 2020). Overall, the threshold values of the different datasets are broadly similar to those of the original Munro dataset.

The Hazard Evaluation Support System (HESS) Integrated Platform was developed as a system to support repeated-dose toxicity predictions using a category approach by providing information on toxicity, metabolism, and possible mechanisms of analog substances (Sakuratani et al., 2013). It contains highly reliable repeated-dose toxicity test data of hundreds of industrial chemicals *via* oral administration, mostly obtained from the existing chemicals survey program under the Chemical Substances of Control Law (CSCL) in Japan. The toxicity database consists of chemical information, purity of tested materials, administration period, No Observed (Adverse) Effect Levels [NO(A)ELs], and over 400 findings of the test. These data have not been analyzed for TTC thus far, but represent a valuable data source as this is a publicly available high-quality dataset. It is expected that the reliability of TTC values will be improved by merging these datasets and expanding the chemical space.

In this study, we constructed a new TTC database from HESS toxicity test data, and analyzed the data to verify the TTC values for each Cramer class. Furthermore, by utilizing metabolism and mechanism information provided by HESS, we characterized the properties of substances with low PODs in Cramer Class III and their critical effects. These works complement previous reports of the TTC approach, and increase the confidence level of the thresholds for non-cancer endpoints.

## Materials and Methods

### HESS TTC Dataset

Repeated-dose toxicity study data was obtained from the HESS Integrated Platform (version 3.3) (HESS, 2020). HESS includes test data of toxicity studies conducted under Japan's CSCL existing chemical safety survey program in compliance with standard test guidelines and Good Laboratory Practice (Sakuratani et al., 2013; J-CHECK, 2020; JECDB, 2020). Moreover, HESS includes the test data collected under Japan's Industrial Safety and Health Act (ISHA, 2020) according to OECD TG408 and of dose range-finding experiments for chronic toxicity studies conducted under the National Toxicology Program (NTP, 2020). It also contains toxicity test data reported in peer-reviewed journal articles equivalent or according to the standard test guidelines. Toxicity profiles were developed by experts on regulatory chemical hazard assessment under the CSCL. Moreover, repeated-dose toxicity studies cited in the Screening Information Data Set (SIDS) developed by the Organization for Economic Co-operation and Development (OECD) High Production Volume Chemicals program were also included in this study (OECD, 2020). The criteria for selecting studies were as follows; the test animal was a rat, the route of administration was oral (gavage, dietary, or drinking water), the administration period was 1–3 months (~28–90 days), and the dosage unit was either given as, or convertible to, mg/kg bw/day. Where multiple test information existed for one substance, one study was selected in the order of higher reliability, a longer study duration, and a lower NO(A)EL value, to be more conservative. The following substance groups were excluded according to the criteria for exemptions from TTC approach that have already been reported: inorganic substances, organic and inorganic metals, organic phosphorus compounds, organic silicon compounds, steroids, azoxy compounds, and natural toxins (Munro et al., 1996; Cheeseman et al., 1999; Kroes et al., 2004).

### POD Preparation

POD values were derived and adjusted as described previously (Yang et al., 2017). Briefly, NO(A)EL values were determined by the lowest dose values based on toxicologically meaningful systemic effects. Test animal-specific findings not relevant to humans, such as alpha 2u globulin-dependent nephrotoxic effects in male rats, were not considered as toxic effects (Baetcke et al., 1991). When systemic effects were observed at the minimum dose and NO(A)EL was not established, the LO(A)EL value was identified, and a LOAEL-to-NOAEL conversion factor of 3 was applied. The subacute/subchronic to chronic adjustment factors were determined be 6 for studies with a 28–83 days administration period as recommended in REACH guidance (ECHA, 2012) and 3 for studies with a 84–179 days administration period (Munro et al., 1996).

### Cramer Classification

Once the HESS TTC dataset was prepared, all substances were divided into the three structural classes defined by Cramer et al. (1978). Cramer classification was performed using the Toxtree (version 3.1.0) decision tree “Cramer Rules, Extended Functions” (Toxtree, 2018) and followed by expert review.

### Derivation of TTC Values

To derive TTC values, the 5th percentile of adjusted POD values was calculated using parametric fitting by assuming a lognormal distribution. Fitting was performed using JMP14.3.0 software (SAS Institute Inc., Cary, NC).

### Human Exposure Threshold Values

To derive the human exposure threshold for each Cramer class, the 5th percentile of POD values calculated from the parametric fitting was divided by the uncertainty factor of 100, accounting for interspecies differences (X10) and intraspecies differences (X10) to determine the daily intake per kilogram of body weight [mg(μg)/kg bw/day], because it is believed that the safety factor provides a reasonable margin of safety in translating the results of analysis of the reference database to humans (Munro et al., 1996). The resulting value was then multiplied by a factor of 60, accounting for the average human body weight (kg).

### Chemotype Profiling

Chemical space of the HESS TTC dataset was characterized by publicly available ToxPrint chemotypes (ToxPrint.org, 2020). The use of this method to describe chemical space in chemical inventories and databases has been reported previously (Yang et al., 2015; Richard et al., 2016). The structure data files of the dataset chemicals were prepared using ChemDraw Professional version 19.1.0.8 (PerkinElmer, Inc. Waltham, Massachusetts, U.S.). Fingerprint files were generated using the public ChemoTyper application (version 1.0) (ChemoTyper.org, 2020). In addition, the profiling of the chemical space was demonstrated by Principal Component Projections. Details of this method has been previously published (Yang et al., 2008, 2017, 2020).

## Results

### Characterization of the HESS TTC Dataset

#### Profile by Data Sources

The HESS database (version 3.3) provided 896 subacute or subchronic studies conducted using 733 substances. In cases where multiple study data were available for one substance, only one study was selected in the order of which had more reliability, a longer duration, or lower NO(A)EL values, as described in the Materials and Methods. As a result, 733 studies were extracted for a total of 733 substances. Then, as previously described, 77 substances were excluded based on the criteria of exclusion of chemical groups. As a result, a HESS TTC dataset consisting of 656 substances covering 656 critical studies was obtained. The initial sources included CSCL Japan, NTP, OECD, SIDS, literature articles of peer-reviewed journals, and ISHA Japan, in the order of number of studies. Subacute studies (28–83 days) and subchronic studies (84–179 days) accounted for 74 and 26% of all studies selected, respectively (Table 1).

**Table 1:** Data sources of the HESS TTC dataset.

**Data source**	**Study duration**		**Total**
	**28–83 days**	**84–179 days**	
CSCL Japan	431	0	431
NTP	4	121	125
OECD SIDS	43	32	75
Journal	10	4	14
ISHA Japan	0	11	11
Total	488	168	656

#### Profile by Chemical Space

Compound overlap was examined between the HESS dataset and previously reported TTC datasets. The Munro dataset contained 613 substances, mostly chemicals and pesticides (Munro et al., 1996), of which 82 substances overlapped with the HESS dataset, while 88% (574/656) substances in the HESS dataset were unique compared to the Munro dataset. Similarly, the COSMOS dataset contained 552 cosmetics-related substances (Yang et al., 2017), 72 of which overlapped with the HESS dataset, and 89% (584/656) of which were unique. The RepDose TTC dataset mostly consisted of industrial chemicals (490 substances) (Tluczkiewicz et al., 2011), 106 of which were shared, and 84% (550/656) of which were unique to the HESS dataset. Moreover, 632 out of 656 substances in the HESS dataset were unique compared to the dataset of OpenFoodTox chemicals (329 substances) (Reilly et al., 2019).

**Figure 1:**
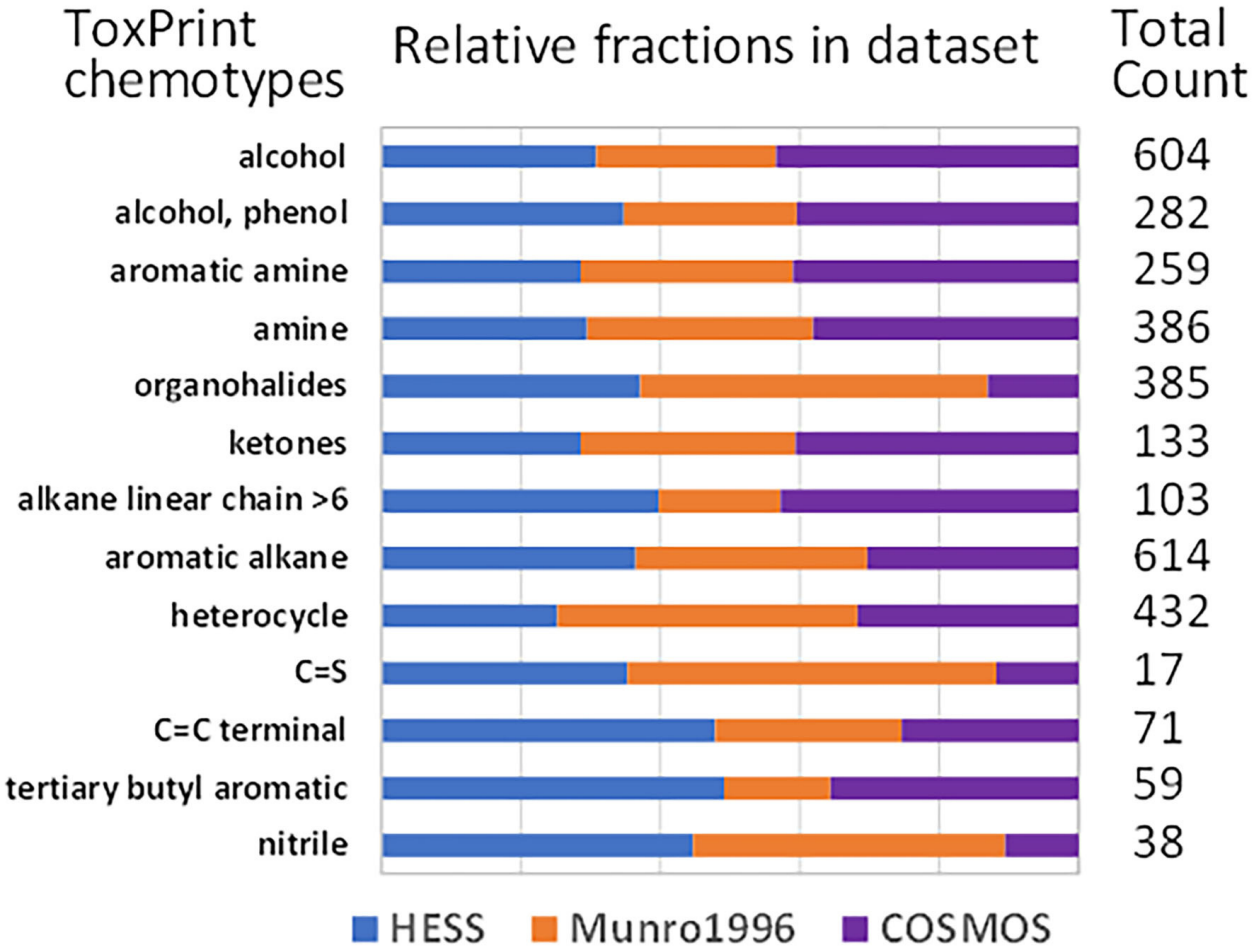
Histogram of ToxPrint chemotypes of the chemicals in HESS, Munro et al. (1996), and COSMOS TTC datasets.

The chemical space of HESS, Munro, and COSMOS substances was characterized by ToxPrint chemotyping (Figure 1). Alcohols, phenols, aromatic amines, amines, ketones, aromatic alkanes, and heterocycles had similar composition ratios among all three datasets. The Munro dataset has fewer structures of alkane linear chains >6 compared to the HESS or COSMOS datasets. The COSMOS dataset had a lower composition rate of organohalides and nitriles than the other two datasets. The HESS dataset contained more carbon-carbon double bond at the terminus of molecules (C=C terminals) and tertiary butyl aromatic compounds, both of which are often found in industrial chemicals. Overall, the HESS dataset had a relatively uniform composition ratio for these chemotypes.

**Figure 2:**
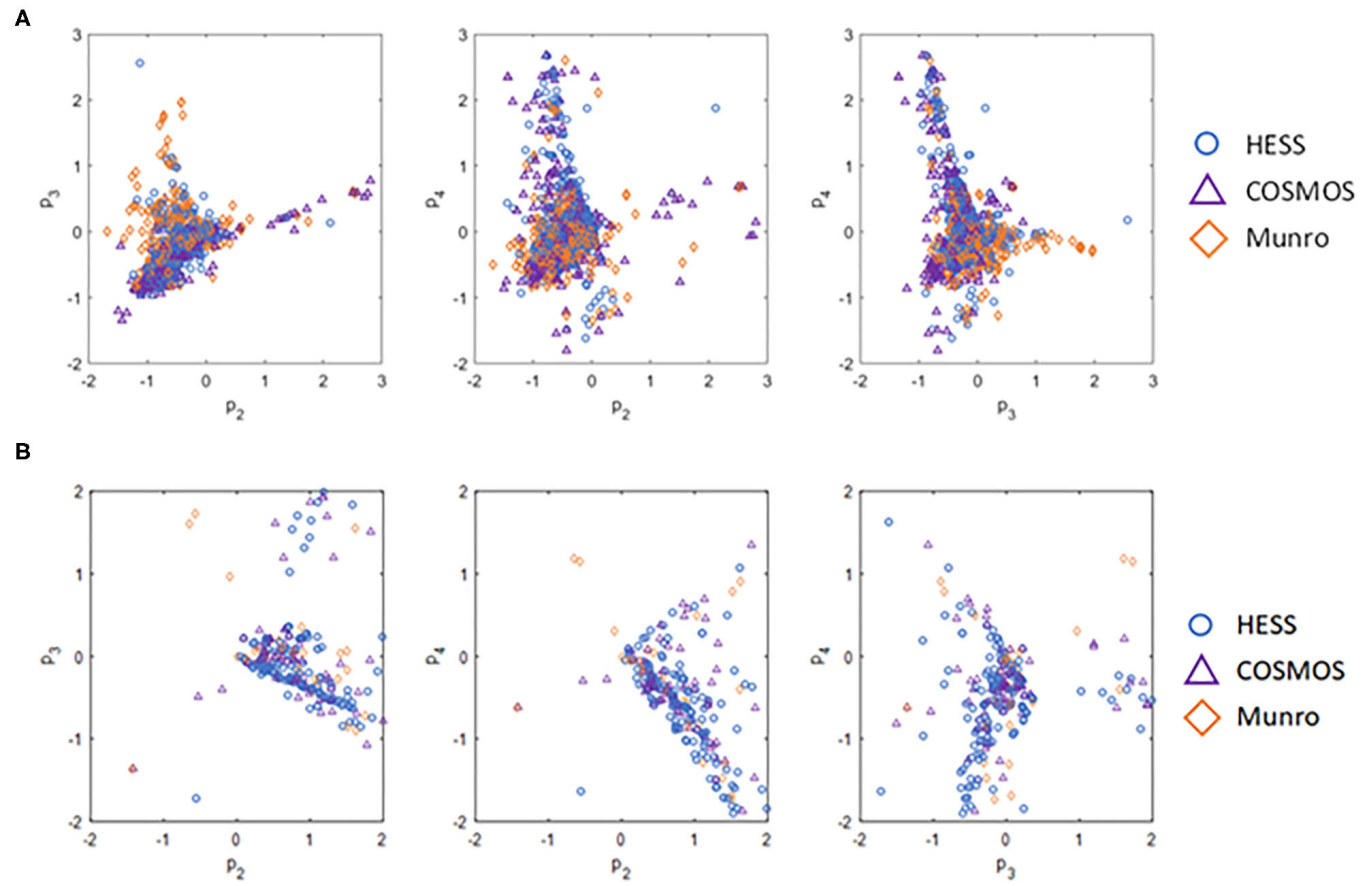
Comparison of **(A)** chemical space of all substances and **(B)** chemical space of Cramer Class I substances of various TTC datasets based on principal component projections.

Chemical space differences between the datasets were also compared using the ToxPrint chemotypes as latent variables in the principal component projections. Figure 2 illustrates the unique chemical space of the HESS TTC dataset.

#### Profile by Cramer Classification

The Cramer classification system was applied to classify each substance in this dataset into one of three classes. Briefly, three Cramer classes are defined as follows (Cramer et al., 1978):

Class I: Substances of simple chemical structure with efficient metabolism and innocuous end products which suggest a low order of oral toxicity.

Class II: Substances that are intermediate. They possess structures that are less innocuous than those in Class I, but they do not contain structural features that are suggestive of toxicity like those in Class III.

Class III: Substances with chemical structures that permit no strong initial presumption of safety and may even suggest a significant toxicity.

The results for the HESS TTC-derived dataset (*n* = 656) is shown, along with Munro's dataset (*n* = 613) and the COSMOS dataset (*n* = 552) in Table 2. The HESS TTC dataset contains 444 compounds categorized as Class III compounds (~68%), while Classes I and II accounted for only 190 compounds (29%) and 22 compounds (3%), respectively. In Munro's dataset, Class I and Class II accounted for ~22 and 5% of compounds, while Class III accounted for the majority of compounds (73%). The COSMOS TTC dataset is enriched with cosmetics-related chemicals of Cramer Class I, and thus is well-balanced between Classes I and III (Yang et al., 2017) (Table 2). Moreover, in the RepDose dataset, Class I, Class II, and Class III accounted for 22, 5, and 73% of compounds, respectively (Tluczkiewicz et al., 2011). In the recently published OpenFoodTox dataset, Classes I and II accounted for only 16 and 2% of the compounds, whereas 82% of substances were assigned to Class III (Reilly et al., 2019). While the antimicrobial TTC dataset (Yang et al., 2020) had a high proportion of biocidal pesticides from EFSA, similar to OpenFoodTox, the overall composition was still quite similar to that of the Munro dataset (20, 4, and 76% of compounds in Classes I, II, and III, respectively). The HESS dataset also showed a similar Cramer classification to other previously reported TTC datasets, regardless of differences in chemical space.

**Table 2:** Distribution of Cramer Classes in the HESS, Munro, and COSMOS datasets.

**Cramer class**	**Number of substances**		
	**HESS**	**Munro et al. (1996)**	**COSMOS**
I	190	137	219
II	22	28	40
III	444	448	293
Total	656	613	552

### POD Distribution of the HESS TTC Dataset

#### General Comparisons of POD Distribution

The POD distribution of the whole dataset as well as that of each Cramer Class was compared for HESS, Munro, and COSMOS datasets (Table 3). The median and geometric mean of adjusted POD values of the whole HESS TTC dataset are both 6.1 mg/kg bw/day. These mean values are lower than those of the Munro and COSMOS datasets, because the distribution for Classes I, II, and III in the HESS TTC dataset was shifted to a lower median and geometric mean of POD values compared to those of the Munro and COSMOS datasets. Class I compounds in the HESS dataset were, on average, more potent than those of the Munro and COSMOS datasets. This unique chemical space occupied by HESS chemicals can be visualized using the principal component (PC) projections (Figures 2A,B). HESS chemicals tended to be loaded on the fourth principal component (PC4), whereas the Munro chemical space more strongly populated the orthogonal PC3 (Figure 2A). Another interesting observation was that COSMOS and HESS datasets had similar loadings on PC4, while PC2 was more occupied by COSMOS chemicals. When the PC projection plots were calculated based only on the Cramer Class 1 substances (Figure 2B), the distribution of HESS chemicals, especially on PC2 and PC4, was markedly different than those of COSMOS and Munro chemicals, again indicating the unique nature of the HESS dataset. Overall, the HESS and COSMOS datasets appreciably expanded the chemical space beyond what was initially covered by Munro.

**Table 3:** logPOD distribution of substances in the HESS, Munro 1996, and COSMOS TTC datasets.

**Cramer class**	**HESS**		**Munro et al. (** **1996** **)**		**COSMOS**	
	**Distribution**	**Stat description**	**Distribution**	**Stat description**	**Distribution**	**Stat description**
All	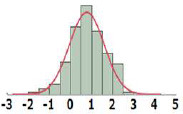	Median^*^:6.1 Geometric Mean^*^:6.1 N:656	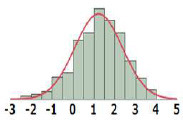	Median:18.0 Geometric Mean:17.2 N:613	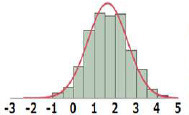	Median:42.2 Geometric Mean:43.2 N:552
I	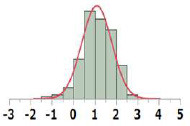	Median:12.0 Geometric Mean:12.5 N:190	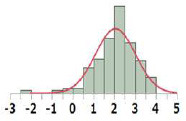	Median:156 Geometric Mean:112 N:137	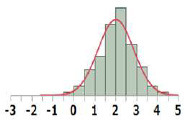	Median:100 Geometric Mean:104 N:219
II	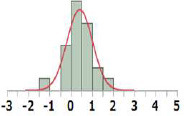	Median:2.5 Geometric Mean:2.6 N:22	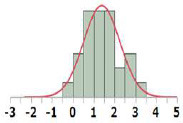	Median:26.5 Geometric Mean:24.4 N:28	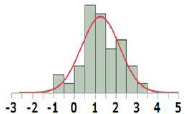	Median:18.3 Geometric Mean:18.5 N:40
III	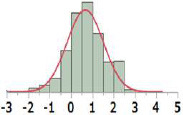	Median:4.9 Geometric Mean:4.7 N:444	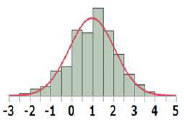	Median:10.0 Geometric Mean:9.5 N:448	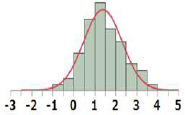	Median:20.7 Geometric Mean:25.1 N:293

* *mg/kg bw/day*.

#### Cumulative Distribution Functions

**Figure 3:**
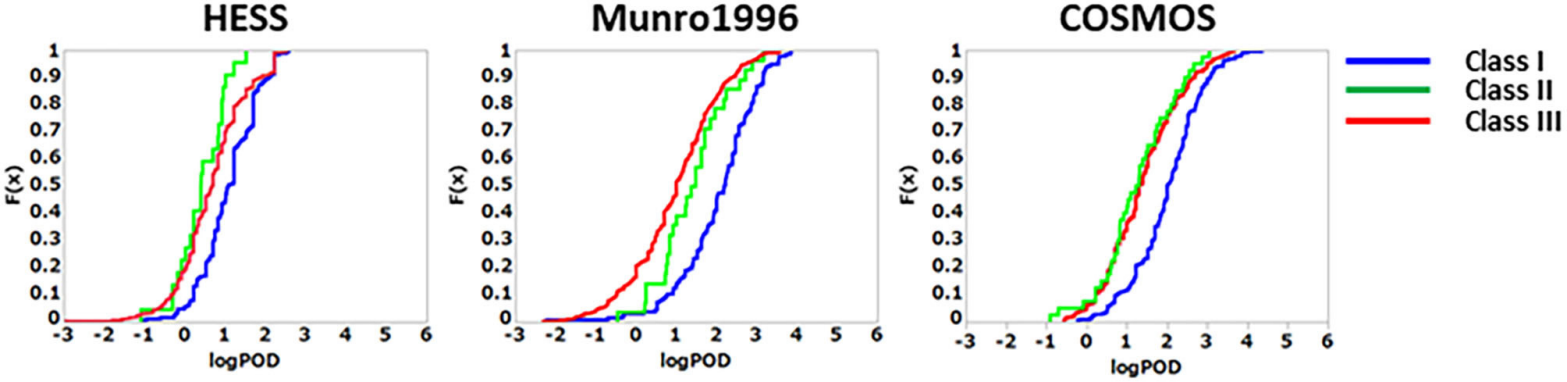
Comparison of the empirical cumulative distribution function in the HESS, Munro et al. (1996), and COSMOS TTC datasets.

To derive TTC values from the HESS TTC dataset and to compare them with those of the Munro and COSMOS TTC datasets, the cumulative distribution of logPOD values was plotted for Cramer Classes I, II, and III (Figure 3). In the Munro dataset, although Classes I and II overlapped at lower POD values, the separation of each Cramer Class is more distinct than that of the HESS and COSMOS datasets, whereas the distributions of Cramer Classes II and III are very similar. Then, the 5th percentile POD was calculated by parametric estimation from the cumulative distribution of the HESS dataset. The 5th percentile POD for Cramer Classes I and III were 0.89 and 0.19 mg/kg bw/day, respectively. Class II has a very small sample size of 22 compared to Class I (190) and Class III (444) in the HESS TTC datasets. The 5th percentile POD value therefore may fluctuate greatly with only a single additional data point in Class II; for this reason, this class was excluded from further analysis.

### TTC Analysis

#### Human Exposure Thresholds of the HESS Dataset and Their Comparisons

The human exposure thresholds for the HESS TTC dataset were determined according to the Munro approach (Munro et al., 1996), by applying a 100-fold safety factor to the 5th percentile POD values (Table 4). Provisional Tolerable Daily Intakes (TDIs) were estimated to be 1.9 and 8.9 μg/kg bw/day for Classes III and I, respectively, which correspond to 111 and 534 μg/person/day for Classes III and I, respectively. No threshold value is proposed for Cramer Class II of the HESS TTC dataset due to limited number of compounds and lack of statistical significance. The TTC values of the HESS dataset were compared with those of Munro and COSMOS datasets expressed in both μg/kg bw/day and μg/person/day (Munro et al., 1996; Yang et al., 2017). In addition, the threshold values of other datasets, RepDose, OpenFoodTox, and antimicrobials were also compared (Tluczkiewicz et al., 2011; Reilly et al., 2019; Yang et al., 2020). The Cramer Class I threshold derived from the HESS dataset was lower than those of these other five datasets, while the Cramer Class III threshold of HESS compounds was similar to those of Munro et al. (1996), RepDose, OpenFoodTox, and antimicrobials, but was lower than that of the COSMOS dataset.

**Table 4:** Comparison of human exposure threshold values.

**TTC dataset**	**Human exposure threshold values**					
	**μg/kg bw/day**			**μg/person/day**		
	**Class I**	**Class II**	**Class III**	**Class I**	**Class II**	**Class III**
HESS	8.9	NA	1.9	534	NA	111
Munro 1996^a^	30	9.0	1.5	1,800	540	90
COSMOS^b^	42	NA	7.9	2,500	NA	470
RepDose^c^	32.2	24.6	1.1	1,930	1,478	63
OpenFoodTox^d^	17	NA	1.5	1,000	NA	87
Antimicrobial^e^	27	4.3	1.2	1,620	258	72

*NA, not applicable,

a *Munro et al., 1996*,

b *Yang et al., 2017*,

c *Tluczkiewicz et al., 2011*,

d *Reilly et al., 2019*;

e *Yang et al., 2020*.

## Discussion

The newly compiled HESS TTC dataset is a high-quality dataset that consists of the reliable subacute or subchronic toxicity data of industrial chemical substances according or equivalent to standard test guidelines. In particular, the toxicity test data under the Japanese CSCL is a new data source that has thus far not been used for non-cancer TTC analysis. As expected, the HESS TTC dataset was found to have less duplication with substances in other reported TTC datasets. This indicates that the HESS TTC dataset is useful for verifying previously reported TTC values, as well as expanding the chemical space.

In the HESS TTC dataset, Class I, Class II, and Class III account for 29, 3, and 68% of chemical substances, respectively. The ratio of the three Cramer classes is similar to those of other TTC datasets. The 5th percentile TDI value of the Cramer Class I chemicals of the HESS TTC dataset was lower than those of the previous datasets (Table 4). The existing chemical survey program of the CSCL has been intended to conduct toxicity tests on substances of toxicological concern for which there is no or limited publicly available toxicity test data, and to use them for risk assessment. Therefore, it is possible that substances with low POD values were preferentially selected. In this class, for instance, some substances with lower POD values have carbon-carbon double bond(s) at the terminals of the molecule and are therefore potentially highly reactive. There are several *tert*-butylphenol derivatives, which are hydrophobic, and therefore possibly slow to be metabolized and excreted. The bias of chemical selection to some extent may contribute to the low threshold value.

There were only 22 substances in Class II of the HESS TTC dataset. Therefore, the addition of a single data point could cause the TTC value to fluctuate wildly, making it difficult to produce a reliable threshold value. In addition, the cumulative curve was almost indistinguishable from that of the Class III. Therefore, no further analysis of the threshold value was performed in this study. Similar results have been reported in other datasets (Yang et al., 2017, 2020; Reilly et al., 2019). The number of Class II chemicals in the respective datasets are 40 (7.2%) in COSMOS, 28 (4.6%) in Munro, 54 (2.8%) in the antimicrobial TTC, and 22 (3.4%) in HESS. Accounting for overlaps, the combined dataset contains a total of 64 (3.4%) unique Class II chemicals, 10 of which are found only in HESS. Inclusion of the new HESS data thus increases the total number of Class II chemicals by nearly 20%.

The Class III threshold value of the HESS TTC dataset (1.9 μg/kg bw/day) was lower than that of the COSMOS dataset enriched with cosmetics-related substances, but was similar to those of the Munro, RepDose, and OpenFoodTox data sets (Table 4). However, the median value of logPOD of the HESS TTC dataset (4.9 mg/kg bw/day) was lower than that of Munro (10.0 mg/kg bw/day) as well as COSMOS (20.7 mg/kg bw/day) (Table 3). This may be due in part to the fact that the HESS TTC dataset, as discussed above, is rich in substances with high toxicity concerns. However, the 5th percentile TDI value was comparable to those of other datasets. The results of this analysis confirm the robustness of Munro's 5th percentile of TDI threshold value.

Inorganic substances, inorganic and organic metals, organophosphorus compounds, and natural toxins were excluded from this analysis. There were 18 organosilicon compounds among those organic metals. The range of provisional TDI values was from 16.7 to 1,670 μg/kg bw/day, which is higher than the Class III threshold values of the HESS as well as the Munro data sets. Thus, it may be possible to apply the non-cancer TTC approach to this class of substances by evaluating further toxicity datasets of organosilicons (Yang et al., 2017).

**Figure 4:**
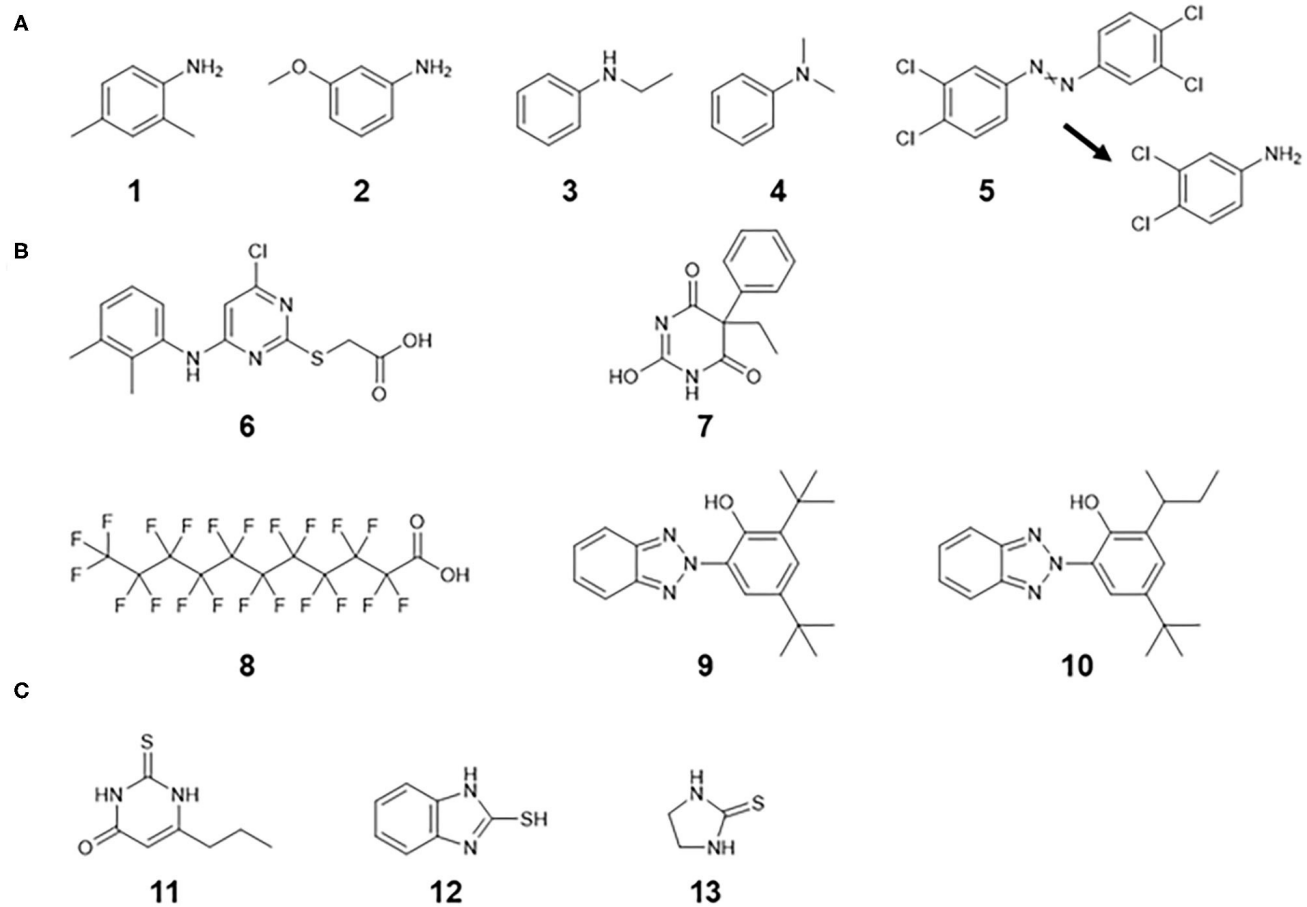
Chemical structures with TDI values below the 5th percentile threshold value of Class III. **(A)** Aniline derivatives causing hemato- and/or hepatotoxic effects. Compound **5** is converted to 3,4-chloroaniline *via* reductive metabolism. **(B)** Hepatic nuclear receptor activators causing hepatic effects. Compounds **6** and **7** are considered to have high activation potency. Compounds **8**, **9**, and **10** are likely to accumulate. **(C)** Thiourea derivatives causing follicular cell hypertrophy in the thyroids by inhibiting thyroperoxidase activity. Compound **1**: 2,4-Dimethylaniline, **2**: 3-Methoxybenzenamine, **3**: N-Ethylaniline, **4**: N,N-Dimethylaniline, **5**: 3,3′,4,4′-Tetrachloroazobenzene, **6**: WY-14,643, **7**: Phenobarbital, **8**: Perfluoroundecanoic acid, **9**: 2-(2′-Hydroxy-3′,5′-di-tert-butylphenyl)benzotriazole, **10**: 2-(2H -Benzotriazol-2-yl)-4-(tert -butyl)-6-(sec -butyl)phenol, **11**: 6-n-Propyl-2-thiouracil, **12**: 2-Mercaptobenzimidazole, **13**: 2-Imidazolidinethione.

There were 22 substances with TDI values below the 5th percentile threshold value of Class III. Among them, four substances were aniline derivatives, which exhibit hematotoxicity and hepatotoxicity (Figure 4A). A possible hemolytic mechanism is involved in metabolic activation to the corresponding N-hydroxylamines (Blaauboer and Van Holsteijn, 1983; Harrison and Jollow, 1987). Class III contained 20 aniline analogs, most of which have known hemato- or hepato-toxic effects as critical effects. Four out of 20 (20%) anilines had TDI values of less than the 5th percentile value.

In addition, substances with a TDI value of less than the 5th percentile showed no structural similarity, some of which may activate nuclear receptors directly or indirectly in the liver to induce hepatocyte hypertrophy (WY-14,643, Phenobarbital, perfluoroundecanoic acid, and two phenolic benzotriazoles shown in Figure 4B) (OECD, 2017; Perkinson et al., 2019). Thioureas in Figure 4C are known to inhibit thyroperoxidase, which is involved in the biosynthesis of thyroid hormones and causes follicular cell hypertrophy in the thyroid (Costa, 2019). This class of chemicals also had low TDI values, but the thyroid toxicity of thioureas is considered less relevant to humans due to the presence of high-affinity thyroid hormone-binding globulin (Costa, 2019). In this study, an uncertainty factor of 100 was applied to derive the TDI values from the corresponding POD values, but it may be necessary to refine the uncertainty factors in consideration of the extrapolation of possible toxicity mechanisms to humans. Another TTC value has been proposed for organophosphates, as they have lower TDI values even than Cramer Class III (Kroes et al., 2004). For these biologically active structures with specific mechanisms or modes of action, a new proposed approach based on structure categories (Yang et al., 2020) may be considered.

In summary, using a new non-cancer TTC dataset, we have expanded the chemical space of TTC datasets, improving confidence of the threshold value for systemic endpoints, as well as contributed to the accumulation and sharing of knowledge on the TTC approach.

## Data Availability Statement

The original contributions presented in the study are included in the article/Supplementary Materials, further inquiries can be directed to the corresponding author.

## Author Contributions

TY developed the manuscript concept, compiled and analyzed the data, and wrote the manuscript. MK carried out the data processing and calculations. AH helped interpret the data and results. CY participated in curation, analysis of the data, and the manuscript preparation. JR assisted in statistical analysis methods and manuscript preparation. All authors contributed to the article and approved the submitted version.

## Conflict of Interest

CY and JR were employed by the Molecular Networks GmbH. The remaining authors declare that the research was conducted in the absence of any commercial or financial relationships that could be construed as a potential conflict of interest.

## Supplementary Material

Supplementary Data File 1 (1) HESS TTC dataset used in this study, (2) original dataset for Figure 1, (3) original dataset for Figure 2, (4) original dataset for Figure 3, and (5) chemical and toxicological information on organosilicons in the HESS Integrated Platform (version 3.3).

## Abbreviations

COSMOS: Integrated *in silico* Models For the Prediction of Human Repeated Dose Toxicity of COSMetics to Optimize Safety
CSCL: Chemical Substances of Control Law
ECHA: European Chemicals Agency
EFSA: European Food Safety Authority
ELINCS: European List of Notified Chemical Substances
HESS: Hazard Evaluation Support System
ISHA: Industrial Safety and Health Act
J-CHECK: Japan Chemicals Collaborative Knowledge Database
JECDB: Japan Existing Chemical Database
LO(A)EL: Low Observed (Adverse) Effect Level
NO(A)EL: No Observed (Adverse) Effect Level
OECD: Organisation for Economic Co-operation and Development
POD: Point of Departure
SIDS: Screening Information Data Set
TDI: Tolerable Daily Intake
TTC: Threshold of Toxicological Concern.
